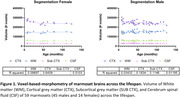# MRI Assessment of Healthy Aging Trajectories in the Marmoset Brain

**DOI:** 10.1002/alz.090970

**Published:** 2025-01-09

**Authors:** Diego Szczupak, Rebecca Bhik‐Ghanie, Bei Zhang, Daniel Papoti, Vinicius Campos, Lauren R Dubberley, T Kevin Hitchens, Fang‐Cheng Yeh, Lauren K Hayrynen Schaeffer, Gregory W Carter, Stacey J Sukoff Rizzo, David J Schaeffer, Afonso C Silva

**Affiliations:** ^1^ University of Pittsburgh School of Medicine, Pittsburgh, PA USA; ^2^ Universidade Federal do ABC, Sao Bernardo do Campo, Sao Paulo Brazil; ^3^ Universidade de Sao Paulo, Sao Carlos, Sao Paulo Brazil; ^4^ The Jackson Laboratory, Bar Harbor, ME USA

## Abstract

**Background:**

The common marmoset (Callithrix jacchus) is an important animal model in neuroscience and neurological diseases (e.g., Alzheimer’s disease ‐ AD), as they present primate‐specific evolutionary features such as an expanded frontal cortex. We established a new consortium with funding support from the National Institute on Aging to generate, characterize, and validate MArmosets as Research MOdels of AD (MARMO‐AD). This consortium develops and studies gene‐edited marmoset models carrying genetic risk for AD, comparing them against wild‐type aging marmosets from birth throughout their lifespan, using non‐invasive longitudinal assessments. Here, we aim to characterize healthy aging trajectories of regional brain volume in a population of marmosets.

**Methods:**

We imaged a cohort of 59 marmosets (45 males, 14 females) across the lifespan (8 to 150 months) using a dedicated 9.4T 30cm bore MRI scanner (Bruker BioSpin Corp, Billerica). The animals were anesthetized under isoflurane and maintained under normal physiological conditions. High‐resolution (250 µm isotropic) T1‐, T2‐, and diffusion‐weighted structural MRI were acquired. The brain images were aligned and registered to the Marmoset Brain Mapping V3 template. The brain was segmented into cortical (CTX) and subcortical (SUB CTX) grey matter, white matter (WM), and cerebral spinal fluid (CSF), and voxel‐based‐morphometry was used to quantify regional brain volume in the left and right hemispheres.

**Results:**

We discovered a decrease in grey matter volume in both males and females with age (Figure 1), reflected by the reduction in several cortical and subcortical brain regions in both sexes. Overall, we discovered that age affects female marmoset brains (38 CTX and 5 SUB CTX regions) more than males (15 CTX and 2 SUB CTX regions). We found no significant age‐dependent changes in WM and CSF.

**Conclusions:**

Our work is the first to thoroughly describe the normal aging of the marmoset brain, a valuable model for age‐related neuropathologies (e.g., AD). This research will establish normative baselines for changes in regional marmoset brain volume with aging that will be used to evaluate our genetically engineered marmoset models of AD, which is the goal of the MARMO‐AD consortium.